# 

**Published:** 1886-12

**Authors:** 


### BOOKS RECEIVED.

*How We Treat Wounds Today.* By Robert T. Morris, M.D. New York: G. P. Putnam's Sons. Chicago: A. C. McClurg & Company. Second edition.

*A Treatise of the Principles and Practice of Medicine.* By Austin Flint, M.D., LL.D. Philadelphia: Lea Brothers & Company. Chicago: A. C. McClurg & Company.

*Massage as a Mode of Treatment.* By William Murrell, M.D., F.R.C.P. Philadelphia: C. Blakiston, Son & Company. Chicago: W. T. Keener.

*Transactions of the American Surgical Association.* Volume IV. By J. Ewing Mears, M.D. Philadelphia: P. Blakiston, Son & Company. Chicago: W. T. Keener.

*The Curability of Insanity.* By Pliny Earle, A.M., M.D. Philadelphia: J. B. Lippincott & Company. Chicago: A. C. McClurg & Company.

*The Functions of the Brain.* By David Ferrier, M.D., LL.D. New York: G. P. Putnam's Sons. Chicago: A. C. McClurg & Company.

*A Laboratory Guide in Urinalysis and Toxicology.* By R. A. Witthans, A.M., M.D. New York: William Wood & Company. Chicago: W. T. Keener.

*Eighth Annual Report of the State Board of Health of Illinois.*

*Thirteenth Annual Report of State Board of Health of Michigan.*

*Seventh Annual Report of the State Board of Health, Lunacy, and Charity of Massachusetts.*

### INDEX TO VOLUME LIII.

PAGE.

**A**

Abdominal Section for Pelvic Abscess ........ 146
Abscess, Hepatic ........ 193
Discussion ........ 291
Actinomycosis Hominis, a Case of ........ 354
Actinomycosis in Man ........ 604
Actinomycosis, Report of a Case of ........ 1
Discussion ........ 54
Agorophobia ........ 600
Alopecia Prematura ........ 478
American Academy of Medicine ........ 517
Anatomy, Practical Review ........ 190
Anatomy, Practical Human Review ........ 424
Anchylosotoneum Duodenale ........ 615
Andrews Edmund, Evacuator for Litholopaxy ........ 610
Anesthesia in General Medicine and Surgery, Review ........
Auscultation, Manual of, Review 425
Autopsies, Manual of, Review ........ 418

**B**

Bacillus Tuberculosis ........ 579, 97
Bacteriology, Review ........ 421
Bartlett, John, Dermoid Cyst ........ 629
  Placenta Prævia ........ 629
  Porro's Operation ........ 211
  Discussion ........ 486
Baths, Hot Water, in Puerperal Peritonitis ........ 182
Belfield, W. T., Cystotomy ........ 379
  Anchylosotoneum Duodenale ........ 615
Billings, Frank, Practice in Vienna ........ 446
  Discussion ........ 480
Bishop, R. W., Alopecia Prematura ........ 478
Brain, Blot Upon, Review ........ 419
Bright's Disease, Review ........ 519
British Medical Association ........ 459
Bubo, Subcutaneous Treatment of ........ 201
  Discussion ........ 296

PAGE.

Byford, H. T., Pelvic Hæmatoceles ........ 317
  Retroflexion ........ 264

**C**

Cæsarian Operation ........ 641
Caldwell, Charles, Hernia of the Umbilical Cord ........ 618
  Letter ........ 369
  Letter ........ 655
Caries ........ 441
Casselberry, W. E., Naso-Pharyngeal Surgery ........ 333
  Galvano-cautery ........ 468
Cataract, Zonular ........ 49
Chicago Gynæcological Society.
  Meeting April 23, 1886 ........ 618
  " May 28, " ........ 146
  " June 18, " ........ 299
  " July 16, " ........ 391
  " Aug. 20, " ........ 485
  " Sept. 17, " ........ 494
Chicago Medical Society.
  Meeting May 3, 1886 ........ 606
  " " 17, " ........ 610
  " June 7, " ........ 163
  " " 21, " ........ 244
  " July 6, " ........ 260
  " " 19, " ........ 275
  " Aug. 2, " ........ 291
  " " 16, " ........ 370
  " Sept. 6, " ........ 381
  " " 20, " ........ 459
  " Oct. 4, " ........ 475
  " " 18, " ........ 478
  " Nov. 1, " ........ 54
Chicago Society of Ophthalmology and Otology.
  Meeting June 8, 1886 ........ 60
  ". Oct. 12, " ........ 65
Cleft Palate and Harelip ........ 606
Clevenger, S. V., Medico-Legal Cases ........ 656
Cocaine Intoxication ........ 74
Colon, Dilatation of ........ 381
Comparative Anatomy and Physiology, Review ........ 190
Correspondence, Foreign ........ 641, 648, 360

PAGE.

Correspondence, Domestic.... ..................656, 663, 182
Crede, Professor, Clinic, in Leipsic.................. 648
Cutaneous Memoranda, Review 187
Cystotomy, Supra-Pubic...... 379

D

Darnall, C. F., Hysteric Trance 28
Davis, N. S., Impressions of the British Medical Association. 459
Dental Malformations in Zonular Cataract............... 49
Dermatology, Recent Progress in....................... 184
Dermoid Cyst Complicating labor...................... 618
De Wolf, Oscar C., State Medicine..................... 4
Dietetics, Review............ 525
Digits, Supernumerary....... 152
Dipsomania and Forensic Medicine..................... 207
Diseases of Infancy and Childhood, Review........... 414
Drainage for Health, Review.. 529

E

Earle, Chas. Warrington..432, 475
Editorial—
Actinomycosis in Man...... 604
The Daily Press and Medical Societies............... 605
Impulsive and Partial Insanity..................... 144
Frank Hastings Hamilton, M. D., LL.D............... 240
The Neurological Review... 241
Ligation of the Vertebral Arteries in Epilepsy......... 241
Asepsis versus Antisepsis... 356
Actinomycosis.............. 360
A Possible New Field in Surgery.................. 454
International Medical Congress.................... 458
Mississippi Valley Medical Association............. 458
Pasteurism................ 46
The Tubercle Bacillus...... 48
Zonular Cataract and Dental Malformations.......... 49
Electric Current, Injury from. 429
Epileptic Insanity........... 648
Epilepsy, Ligation of the Vertebral Arteries in (Editorial). 241
Gray.................... 282
Ergot in Uterine Fibroids..... 153

PAGE.

Etheridge, James H., Hysterectomy...................... 495

F

Favus, a Clinical Lecture...... 344
Fibroids, Uterine, treated with Ergot.................... 153
Fitz-Gerald, F. W., Hot-baths. 183
Forwood, W. H ........... 125

G

Galvano-Cautery in Pharyngeal and Nasal Surgery.......... 333
Gudden, Professor v.—Notice of death of............... 606
Gradle, H., Pure Drinking Water.................. 606
Gray, J. L., Ligation of the Vertebral Arteries............ 282

H

Hæmatoceles, Pelvic ......... 317
Discussion............... 396
Hamill, Robert C., Obituary... 299
Hamilton, Frank Hastings.240, 381
Hamilton, John B., Inguinal Hernia .................. 371
Hare Lip and Cleft Palate .... 606
Headache from Defective Vision.................... 475
Helm Scott, Treatment of Bubo 201
Discussion .............. 296
Hepatic Abscess.............. 193
Discussion .............. 291
Hernia, Abstract ............ 411
Hernia, Inguinal, Radical Cure of...................... 371
Hernia of the Umbilical Cord. 618
Hirschsprung, Professor...... 68
Hoadley, A. E., Rectal Stricture 18
Discussion............... 52
Houghton, A. S., Danger in Specialism ............... 181
How We Treat Wounds To-Day, Review.............. 532
Hydrophobia and Imagination 125
Hysterectomy ............494, 506
Hysteric Trance.............. 28

I

Ichthyol................... 32
Insanity and Its Treatment, Review .................. 669
Insanity a Symptom of Bodily Disease.................. 661
Insanity, Impulsive and Partial 144
Intubation of the Larynx..132, 244
International Medical Congress 457
Circular No. 2............ 324
Italian Surgical Congress ..... 641

PAGE.

J

Jackson, A. Reeves, Intra-uterine stem 163
Jaggard, W. W., Gravid uterus with Adnexa 299
Tænia Mediocanellata 616
Jewell, J. S., Dilatation of the Colon 381

K

Knee, Resection of 643
Kossakowski, M. P., Hare-lip 606

L

Labour, Impediment to 160
Lagoria, A., Letter 643
Litholopaxy, Evacuator for 610
Lyford, W. H., Scleroderma 609

M

Mac Arthur, L. L., Hepatic Abscess 193
Discussion 291
Mann, Edward C., Dipsomania 207
Medicine, Study of, Review 424
Membranous, Cast of the Trachea 370
Michigan State Medical Society 616
Mississippi Valley Medical Association 458
Moyer, Harold N., Electric Current 429

N

Nasal Catarrh, Review 672
Nervous Diseases, their Symptoms and Treatment, Review 670
Neurological Review, The 241

O

Obituary, Professor v. Gudden 606
Ochsner, A. J., Actinomycosis, 1
Discussion 54
Onanism in Young Children 68
Os Uteri, Occlusion of 160
Discussion 396

P

Pachymeningitis, Cervical 332
Paranoia 656
Park, Augustus V., Railway Injury 440
Parkes, Charles T., Uterine Fibroids 153
Urinary Fistula 261
Hysterectomy 506
Pasteurism 46
Pasteur's Laboratory 432
Pathology, General Review 522

PAGE.

Pelvic Abscess, Abdominal Section for 146
Peritonitis, Puerperal 183
Pessary Stem 163
Porro's Operation 211
Discussion 487
Practical Medicine, Review
Puerperal Convalescence, Review
Pupil, Peculiar Form of Motor Disturbance of 76
Pure Drinking Water 607

R

Reynolds, Henry J., Progress in Dermatology 184
Favus 344
Rectum, Stricture of 18
Discussion 52
Rhinitis Sympathetica 42
Reviews and Book Notices.
Insanity and its Treatment, Blandford 669
Treatise on Nervous Diseases, Webber 670
Diseases of the Spinal Cord, Bramwell 671
Nasal Catarrh, Robinson 672
The Student's Manual of Venereal Diseases, Hill 187
A Manual of Diseases of the Skin, Squire 187
Cutaneous Memoranda, Piffard 187
Venereal Memoranda, Morrow 188
Comparative Anatomy and Physiology, Bell 190
Practical Anatomy, Heath 190
Puerperal Convalescence, Kucher 321
Diseases of the Digestive Organs in Infancy and Childhood, Starr 414
Elements of Surgical Diagnosis, Gould 417
Manuel des Injections, Bourneville 417
Manuel de Technique des Autopsies, Bourneville 418
The Blot upon the Brain 419
Traite Elémentaire d'Anatomie, Médical du Systeme Nerveux, Fere 419
A Manual of Therapeutics, Waring 420
Medical and Surgical Directory of the United States 420

PAGE.
The Methods of Bacteriological Investigation, Hueppe 421
Essentials of Vaccination, Hardaway 422
The Best Preliminary Education for the Study of Medicine, Stevens 423
Practical Human Anatomy, Weisse 424
Manual of Auscultation, Flint 425
Manual of Operative Surgery, Stimson 426
Bright's Disease, Purdy 519
Introduction to General Pathology, Sutton 522
Principles and Practice of Surgery, Hamilton 523
A Manual of Dietetics, Fothergill 525
Handbook of Practical Medicine 527
Drainage for Health 529
How We Treat Wounds To-Day, Morris 532
Local Anesthesia, Corning 77
A Manual of Surgery, Treves 79
Treatise on Medicine, Bartholow 80
Healing of Arteries After Ligature, Warren 81
How We Treat Wounds To-Day, Morris 83
Differential Medical Diagnosis, Cutler 83
Lectures on Dietetics and Dyspepsia, Roberts 83
Dictionary of Practical Surgery, Heath 84
Massage as a Mode of Treatment, Murrell 85
A Manual of Minor Surgery, Heath 86
A Treatise on the Practice of Medicine, Flint 87
An Atlas of Clinical Microscopy, Peyer 89

S

Schirmer, A., Actinomycosis 354
Schmidt, H. D. 97, 579
Scrotal Tumors, Diagnosis of 275
Senile Gangrene 644
Sinclair, Charles F., Defective Vision 475
Skin, Diseases of, Review 187
Small, A. R., Case of Pistol-shot Wound 179
Specialism, Danger in 181

PAGE.
Spinal Concussion 659
Spinal, Cord Diseases of, Review 671
Spleen, Extirpation of 645
State Medicine 4
St. Bartholomew's Hospital Reports, Abstract 405
Steele, D. A. K., Scrotal Tumors 275
Stricture of the Rectum 18
Discussion 52
Sub-cutaneous Injections, Review 417
Surgery, a Manual of, Review
Surgery, New Field in 454
Surgery, Operative Review 426
Surgery, Review 523
Surgical, Diagnosis, Review 417

T

Tait, Lawson 146
The Daily Press and Medical Societies 605
Therapeutics, Manual of, Review 420
Thyroid Gland, Extirpation of 641
Tracheotomy, Intubation as a Substitute for 132, 244
Trance, Hysteric 28
Tuberculosis, Bacillus of 97, 579
Tucker, James I., Undiagnosable Maladies 613

U

Umbilical Cord, Hernia of 618
Undiagnosable Maladies 613
Urinary Fistula 261
Uterine Flexions, Stem Pessary in 163
Uterus, Retroflexion of 264

V

Vaccination, Review 422
Van Horn, A. K., Agorophobia 600
Venereal Diseases, Review 187
Venereal Memoranda, Review 188
Vertebral Arteries, Ligation of in Epilepsy, Editorial 241
Gray 282

W

Waxham, F. E. 132
Occlusion of the Os Uteri 160
Intubation 244
Specimen 370
Wines, Fred H. 668

Z

Zeisler, Joseph, Ichthyol 32

202

Index to new pagination

SUBSCRIPTION PRICE, $3.00 PER YEAR, IN ADVANCE.

Whole Number, 323 DECEMBER, 1886. Vol. LIII. Number 6.

LIBRARY
INDEXED
S.G.O.

INDEX
MEDICUS

THE

### CHICAGO MEDICAL JOURNAL AND EXAMINER

EDITORS:

S. J. JONES, M.D., LL.D. HAROLD N. MOYER, M.D.

COLLABORATORS.

CHICAGO:

PROFESSOR J. A. ALLEN, M. D., LL. D.
PROFESSOR E. ANDREWS, M. D., LL. D.
PROFESSOR JOHN BARTLETT, M. D.
PROFESSOR W. H. BYFORD, A. M., M. D.
PROFESSOR O. C. DEWOLF, A. M., M. D.
PROFESSOR E. C. DUDLEY, A. B., M. D.
PROFESSOR J. H. ETHERIDGE, A. M., M. D.
PROFESSOR C. FENGER, M. D.
PROFESSOR J. N. HYDE, A. M., M. D.
PROFESSOR E. F. INGALS, A. M., M.D.
PROFESSOR W. W. JAGGARD, A. M., M. D.
PROFESSOR H. A. JOHNSON, M. D., LL. D.
PROFESSOR CHARLES GILMAN SMITH, A. M., M.D.

MILWAUKEE, WISCONSIN:
PROFESSOR N. SENN, M. D.

WASHINGTON, D. C.:
S. O. RICHEY, M. D.

UNITED STATES ARMY:
SURGEON, D. L. HUNTINGTON, M. D.

UNITED STATES NAVY:
MEDICAL DIRECTOR, J. M. BROWNE, M. D.
MEDICAL DIRECTOR, A. L. GIHON, A. M., M. D.

